# Goal Pursuit in Individuals with Chronic Pain: A Personal Project Analysis

**DOI:** 10.3389/fpsyg.2016.00966

**Published:** 2016-06-28

**Authors:** Geert Crombez, Emelien Lauwerier, Liesbet Goubert, Stefaan Van Damme

**Affiliations:** ^1^Department of Experimental-Clinical and Health Psychology, Ghent UniversityGent, Belgium; ^2^Center for Pain Research, University of BathBath, UK; ^3^Department of Health and Well-being, University Colleges Leuven-LimburgLeuven, Belgium

**Keywords:** chronic pain, goals, catastrophizing, problem solving, acceptance

## Abstract

**Objectives:** In individuals with chronic pain (ICPs), controlling pain often is a salient goal, despite the difficulty to achieve it. This situation may bring along frustration and distress. Yet much remains unknown about the content, appraisal, and structure of goals that ICPs pursue. Here, we explore these goals, and specifically focus upon possible differences and interrelations between pain control goals (e.g., “to control my pain”) and non-pain goals (e.g., “to go to work”).

**Design and Methods:** “Personal Project Analysis” was used in 73 ICPs (48 females; 25 males; *M*_age_ = 49.85 years; *SD* = 9.72) to elicit goals and goal appraisals. Interrelations between pain and non-pain goals, namely interference (i.e., negative influence), facilitation (i.e., positive influence), and necessary condition (i.e., conditional relation between pain control goal and non-pain goals) were measured with three items. Self-report measures of pain intensity, pain catastrophizing, problem solving and acceptance were completed.

**Results:** Participants reported a variety of goals. Appraisals of pain control goals were less favorable than appraisals of non-pain goals. ICPs with higher acceptance and meaningfulness of life reported more control over pain goals, and more progress in reaching pain control goals. These individuals also reported an overall much more positive appraisal of non-pain goals (i.e., less stress, difficulty, more progress, control). In contrast, high catastrophizing and the need to solve pain were negatively related to goal appraisals. Importantly, ICP’s with high perceived meaningfulness of life despite pain experienced less necessity to achieve pain control goals in order to achieve non-pain goals. This was opposite for individuals with high levels of catastrophizing.

**Discussion:** An understanding of why ICPs may become stuck in attempts to control their pain does not only require an understanding of how individuals appraise their pain, but also requires an understanding of how pain and non-pain goals interrelate. In particular, the view that controlling pain is necessary in order to be able to achieve other goals seems detrimental.

## Introduction

Some individuals with chronic pain (ICPs) adjust well to their pain. Others report high levels of interference of their daily activities by pain, and experience distress and despair (McCracken and Eccleston, 2003, 2005; Viane et al., 2003). Research has attempted to understand why these individuals are stuck in a vicious circle of enduring pain, disability and suffering. It has been proposed that distress and suffering result from a rigid search for a solution with regard to an experience that (unfortunately) cannot be controlled (McCracken and Eccleston, 2003; Eccleston and Crombez, 2007). Indeed, medical treatments often cannot provide satisfying pain relief for ICPs. Nevertheless, ICPs may remain searching for a solution, and this search may start to dominate their life at the expense of the pursuit of other valued activities.

Trying to solve the problem of chronic pain, or at least reducing it to an acceptable level, is typically the first response to pain-induced goal disturbance (Eccleston and Crombez, 2007; Van Damme et al., 2008). ICPs may then engage in a variety of behaviors, such as looking up information on the internet, visiting healthcare providers, taking medication, resting in bed, or avoiding pain-inducing activities. A second possible response is accepting the problem of chronic pain as insoluble. ICPs may then disengage from trying to solve the problem of chronic pain, and reengage in other valued goals despite the presence of pain (McCracken and Eccleston, 2003). In line with this view, acceptance has been related to favorable outcomes in the context of chronic pain (McCracken and Eccleston, 2003, 2005; Viane et al., 2003). Problems may arise when ICPs persist in futile attempts to solve or control pain at the expense of the pursuit of other valuable life goals. Such attempts have been dubbed ‘misdirected problem solving’ (Aldrich et al., 2000; Eccleston and Crombez, 2007). In line with this view, attempting to solve the problem of chronic pain has been linked to unfavorable outcomes (De Vlieger et al., 2006; Crombez et al., 2008). Adhering to an agenda of pain control is also believed to more readily occur in those who catastrophize about their pain (De Vlieger et al., 2006), i.e., excessively worry about pain and its possible consequences.

As yet, we do not have a broad understanding of why ICPs may become stuck in attempts to control pain. We also do not have a broad view on how ICPs experience the pursuit of pain control amidst the other goals that often are simultaneously pursued (Crombez et al., 2012). Overall, the assessment of goals in the context of chronic pain is not a well-studied area. Most of these studies focus on the assessment of non-pain goals in chronic pain (Karoly and Ruehlman, 1996; Karoly and Lecci, 1997; Aﬄeck et al., 1998, 2001; Karoly et al., 2008; Hardy et al., 2011), but do not assess how ICPs construct and appraise their pain control goals. Therefore, this study focuses upon pain goals, in particular the goal to control pain, and their interrelations with other, non-pain, goals.

The present study adopts the “Personal Project Analysis” (PPA, Little, 1983) to assess personal goals and their characteristics. According to PPA, personal projects are “extended sets of personally salient action” (Little and Gee, 2007, p. 25). Typically, a PPA requires respondents to list goals that are currently important to them, to rate these goals on a number of dimensions or appraisals, and to report on the interrelationships between the goals (goal structure). For goal structure, we were particularly interested how working on a pain control goal facilitates the pursuit of other goals (goal facilitation: e.g., “to control pain” allows me “to go to work”), or interferes with the progress on other goals (goal interference: e.g., “to control pain” hinders me “to go out with friends”) (Riediger and Freund, 2004). Although the characterization of goal interrelations in terms of facilitation and interference is theoretically well established (Riediger and Freund, 2004), we found that one particular characteristic was lacking. Clinical practice learns that patients often frame their attempts to control pain as a necessary condition to be able to continue with their life (Malec et al., 1977). We believed that this characterization was insufficiently captured under the constructs of goal facilitation and interference (Riediger and Freund, 2004). For that reason, we asked ICPs directly about this feature, which we dubbed “necessary condition” (e.g., “to be able to control pain” is necessary in order “to spend time with family”).

Given the current state of research, we opted for an exploratory approach (Rozin, 2009), in which the content, appraisals and structure of the goals that ICPs pursue, were broadly assessed. We sought answers to 4 questions: (1) What types of goals are spontaneously elicited by ICPs?; (2) How do ICPs appraise their pain control goals, and how does this compare with non-pain goal appraisals?; (3) How do pain control goals relate to other, non-pain goals?; (4) Finally, we were interested in whether the goal appraisals and interrelationships were related to some key constructs involved in misdirected problem solving, such as catastrophic thinking about pain, attempts to solve pain and acceptance.

## Materials and Methods

### Participants

The present study was part of the GPD-I study consisting of three studies on chronic pain and functioning. More information and details about this study can be found on http://hdl.handle.net/1854/LU-3050986. Participants were recruited from Flemish patient associations from December 2010 onward over a 4-month period. Inclusion criteria were (a) being aged between 18 and 65 years, (b) having sufficient Dutch language skills to fill out self-report measures, and (c) having pain for at least 6 months. Exclusion criteria were (a) reporting headache as a primary complaint, (b) reporting a psychiatric disorder (other than pain disorder) as primary complaint, and (c) physical limitations that made it impossible to participate in computer tasks. Three hundred and fifteen ICPs agreed that they could be contacted for the GPD-I study. Of these 315, 267 ICPs were contacted by phone and invited to participate. Eighty-one ICPs were eligible and agreed to participate in the study. However, 7 ICPs refrained from participation owing to health problems, and one participant was excluded because he/she did not report pain at the moment of testing.

The final sample consisted of 73 participants (48 females; 25 males; mean age = 49.85 years; *SD* = 9.72). Most ICPs were married or living together (69.9%), 39.4% had a higher education (longer than the age of 18 years). Only 18.1% was in paid employment or followed education, 7% was in unpaid employment, 13.9% was retired, and 4.2% was unemployed. All others received disablement insurance benefits (55.5%) or were legally trying to receive one (1.4%). The mean pain duration was 14.04 years (*SD* = 9.37). Back pain was the most reported pain location (90.4%). Participants also frequently reported pain in other body sites, such as the legs (67.1%), neck (67.1%), arms (46.6%), and head (32.9%). On average, participants reported pain on at least three different locations (*M* = 3.84, *SD* = 1.88). Socio-demographic information on non-participants was not available. The Local Ethics Committee of the University approved the study protocol.

### Measures

#### Personal Project Analysis

We followed the guidelines of the Personal Project Analysis (Little, 1983) and conducted a semi-structured interview in which clarification, prompts and feedback were provided to elicit goals. Participants were asked to list all their current goals. We asked participants to report as many personal goals as possible that they had for the near future, currently judged to be important, and still expected to be important in the upcoming months. Whenever ICPs did not spontaneously mention a pain control goal during the free-elicitation phase, we required participants to identify one. We asked participants to write down their goals with a few words or short sentences (Ogilvie et al., 2001).

Goals were coded into 12 categories. We followed a standard coding procedure. Two independent raters were asked for the initial coding. Whenever there was disagreement, a third rater was assigned and recoded until consensus was achieved. The coding was based on existing taxonomies of goals (Chulef et al., 2001) and consisted of the following categories: interpersonal goals (e.g., to keep in touch with friends), intrapersonal goals (e.g., to be loving), health/physical domain goals (e.g., to lose weight), work/education goals (e.g., to do voluntary work), financial goals (e.g., to be financially independent), leisure/entertainment-related goals (e.g., to travel more), and psychological/mental well-being goals (e.g., to be full of energy). When we examined the list of goals participants provided, we decided to add three other categories: one related to house-holding (e.g., cleaning the house), one related to exercise (e.g., to walk on a daily basis), and one related to social validation of one’s pain (Hamilton et al., 2005) (e.g., to be believed that the pain is real). Individuals’ pain goal, i.e., the goal to control pain was classified separately as pain control (e.g., to have less pain). Finally, there was a rest category, consisting of all goals that could not be classified into one of the 11 categories above. The inter-rater reliability was high (Cohen’s Kappa = 0.77, *p* < 0.001) and there was an overall simple agreement coefficient of 79.5% (421-86/421).

In line with PPA guidelines (Little, 1983), participants were asked to select their two most important non-pain goals, and their pain control goal. Then, they were asked to rate these goals on a number of appraisal dimensions. A standard set of goal appraisals has been identified (Little, 1989, 1998; Austin and Vancouver, 1996). These usually include the following: meaning (e.g., importance, value congruency, self-identity), structure (e.g., control, time), efficacy (e.g., satisfaction with progress), or stress (e.g., stress, difficulty) (Little and Chambers, 2004). We selected a limited number of dimensions, mainly to avoid mental overload in participants. The following dimensions were included: (1) importance (“This goal is important to me”); (2) difficulty (“I find it hard to achieve this goal”); (3) control (“I feel I am in control of this goal”); (4) stressfulness (“I find it stressful to pursue this goal”); (5) time (“I spend a lot of time in pursuing this goal”); (6) progress (“I am satisfied with the progression I made in achieving this goal”); (7) self-identity (“This goal says a lot about who I am”); and (8) value (“This goal is highly valuable to me”). Each appraisal had to be rated on a 7-point Likert scale, ranging from 0, *not at all*, to 6, *completely*.

Goal interrelationships were measured by three items. Items were formulated during an iterative process, and piloted for comprehensibility with ICPs. Participants were instructed to rate the interrelationships of each possible pair of goals from their selected set of three goals, i.e., their pain control goal [C]) and their two most important non-pain goals [A and B], There were six possible pairs (A-B, B-A, A-C, C-A, B-C, C-B). For each pair of goals, participants rated three items, one item reflecting intergoal interference (e.g., “To what extent does the pursuit of goal C have a negative influence on the pursuit of goal B?”), one item reflecting intergoal facilitation (e.g., “To what extent does goal C have a positive influence on the pursuit of goal B?”), and one item reflecting goal necessity (e.g., “To what extent is it necessary to achieve goal C in order to be able to achieve goal B?”). As such, participants responded to a total of 18 items. All items were rated on a 5-point Likert scale, ranging from 1, *not at all*, to 5, *very much*. Of importance for this study were the goal interrelationships between the pain control goal and the non-pain goals.

#### Questionnaires

Pain severity was measured by means of the two-item pain severity subscale of the Dutch version of the Multidimensional Pain Inventory (MPI; Lousberg et al., 1999) (i.e., “Rate the level of your pain at the present moment”, and “On average, how severe has your pain been during the last week”). Ratings are made on a 7-point scale (from 0 to 6). The sum score of the two items may range between 0 and 12. The MPI has been shown to have good reliability and validity. Test–retest reliability (*r* = 0.71) and Cronbach’s alpha (α = 0.74) of the pain severity subscale are both adequate. Data on construct validity are also adequate (Lousberg et al., 1999). Cronbach’s alpha in this study was α = 0.80.

We used the Pain Solutions Questionnaire (PaSol; De Vlieger et al., 2006) to assess efforts at changing, solving or accepting pain and the problems associated with pain. The PaSol has 14 items grouped into four interrelated scales: (1) solving pain (four items; e.g., “I try everything to get rid of my pain”); (2) meaningfulness of life despite pain (five items; e.g., “Even when I have severe pain, I still find my life meaningful”); (3) acceptance of the insolubility of pain (three items; e.g., “I can live with the idea that there is no solution for my pain”); and (4) belief in a solution (two items; e.g., “I am convinced that there is a treatment for my pain”). Participants are instructed to describe the degree to which each statement applies to them. Each item is answered on a 7-point Likert scale, ranging from 0, *not at all applicable*, to 6, *highly applicable*. Although the original PaSol has demonstrated good reliability and validity (De Vlieger et al., 2006), subscale scores tend to be heavily skewed in ICPs (Crombez et al., 2008). Therefore, we decided to slightly adjust the wording of 13 out of 14 items and formulated them in a more extreme way, i.e., (1) solving pain; e.g., “I would try *really* everything to get rid of my pain”; (2) meaningfulness of life despite pain; e.g., “*Even when in pain*, I still find my life meaningful”; (3) acceptance of the insolubility of pain; e.g., “I can live with the idea that there *exists* no solution for my pain”; (4) belief in a solution; e.g., “I am *truly* convinced that there is a treatment for my pain”. Cronbach’s alpha’s in this study were α = 0.85, 0.86, 0.78, and 0.86, respectively, for the four scales. Subsequent analyses showed that three (i.e., solving pain, acceptance of the insolubility of pain, and belief in a solution) out of the four subscales met criteria for normal distribution.

The Dutch version of the Pain Catastrophizing Scale (PCS-; Sullivan et al., 1995; Crombez et al., 1998) was used to measure catastrophic thinking about pain. It is a 13-item scale for both non-clinical and clinical populations. Participants are asked to reflect on past painful experiences and to indicate the degree to which they experienced each of the 13 thoughts or feelings during pain on a 5-point scale (e.g., “I can’t seem to keep it out of my mind”, or “I become afraid that the pain may get worse”). Scores range from 0 to 4. The PCS has shown to be valid and highly reliable (Osman et al., 2000; Van Damme et al., 2002). Cronbach’s alpha in this study was α = 0.90.

### Procedure

A self-report assessment and a semi-structured interview were employed. First, participants were invited to fill in a set of questionnaires at home. These could be completed online (i.e., Limesurvey, *n* = 62) or on paper (*n* = 11). Next, participants were invited to the university. Participants were further informed about the study and provided a written consent. They were then requested to provide socio-demographic information and completed some brief questions about their pain. Subsequently, participants responded to the Personal Project Analysis through a semi-structured interview format.

### Statistical Strategy

Data were analyzed using the Statistical Package for Social Sciences (SPSS 20.0) and Microsoft Excell 2007 for Windows (Microsoft^®^ Office Excell^®^ 2007). Counting the number of times a participant mentioned at least one goal of a specific category and calculating relative percentages, enabled us to investigate the frequency of goal types reported by our sample.

As we were only interested in the effects of the pain control goal (Goal C) on the other two goals (goal A en B), we averaged the ratings of the two non-pain goals for all further statistical analyses. Descriptive statistics (mean, *SD*) were calculated for each of the goal appraisals of both the pain as well as non-pain goals. Further, a series of pairwise *t*-tests or non-parametric Wilcoxon signed-rank tests was conducted to examine whether there was a significant difference in how ICPs appraised their pain control goal compared to their non-pain goals. To obtain a standardized measure of the magnitude of the observed effects, i.e., a standardized difference between two means, effect sizes (Cohen’s *d*) for independent samples were calculated using Morris and DeShon’s formula (Borenstein et al., 2009). The 95% confidence interval (95% CI) was also calculated. Cohen’s *d* is an effect size that is not design-dependent and conventional norms are available (Field, 2005). We determined whether Cohen’s *d* was small (0.20), medium (0.50), or large (0.80) (Cohen, 1988). Lastly, Pearson correlations or non-parametric Kendall’s tau correlations were calculated to describe the association between goal appraisals on the one hand and problem solving, acceptance, and catastrophizing about pain on the other hand.

In order to examine whether participants reported high levels of pain goal interference, facilitation and necessity, we focused upon the effects of the pain control goal (Goal C) on the other two goals (goal A en B). We calculated descriptive statistics (mean, *SD*), frequencies and proportions of response options across the sample. We used response options ≥4 as indicators of high levels (Riediger and Freund, 2004). Furthermore, Pearson or Kendall’s tau correlations were calculated to assess associations between intergoal variables. Lastly, Pearson or Kendall’s tau correlations were calculated to examine associations between pain control goal interference, facilitation and necessary condition on the one hand and solving pain, acceptance and catastrophizing on the other hand.

## Results

### Type of Goals

Participants listed an average of 5.76 goals (*SD* = 2.01; range 3–12). We found that 41.1% of the participants spontaneously reported at least one pain control goal. Also, participants frequently reported one or more goals in the following life domains: interpersonal (80.82%), work/education (49.32%), leisure time (46.58%), exercise (45.21%), and health/physical well-being (41.10%). The least mentioned were goals related to social validation for one’s pain (6.85%). **Table 1** shows examples of goals reported within each domain.

**Table 1:** Examples of goals provided by participants.

Life domains	Sample goals
Pain Control	To have less pain To live without pain
Interpersonal	To build up social contact To maintain contact with friends
Intrapersonal	To get to know and live with my limitations To be less anxious in contact with other people
Health/Physical Well-being	To lose weight To sleep better
Work/Education	To be able to work again To volunteer in helping students pass their language courses
Finances	To have no financial worries To save money to be able to buy a car
Leisure Time	To travel To do cultural stuff (e.g., concerts, musicals, expositions)
House holding	To clean the house To be able to do the cooking
Psychological/Mental Well-being	To be able to enjoy pleasant things (e.g., watching kids play together) To feel useful again
Exercise	To be able to keep on doing exercise (e.g., swimming, walking) To improve my walking condition
Social Validation	To have others to know what pain is about To be believed by other people
Other	To grow old To wish everybody a good future

### Goal Appraisals

**Tables 2** and **3** display descriptive statistics on goal appraisals. A series of pairwise *t*-tests or non-parametric Wilcoxon-signed rank tests was conducted to examine whether there were significant differences in goal appraisals between one’s pain and non-pain goals. Results indicated that participants rated the pain control goal as more difficult to achieve, *t*(72) = –2.80, *p* = 0.007, Cohen’s *d* = 0.38, 95% CI [0.11, 0.66], and more stressful while pursuing, *t*(72) = –2.09, *p* = 0.04, Cohen’s *d* = 0.27, 95% CI [0.01, 0.52] than the non-pain goals. Participants also reported to spend more time in achieving the pain control goal than the non-pain goals, Z = –2.48, *p* = 0.013, Cohen’s *d* = 0.29, 95% CI [-0.01, 0.58]. Of note, the pain control goal was rated to be related less to one’s identity, *t*(72) = 2.85, *p* = 0.006, Cohen’s *d* = –0.38, 95% CI [-0.65, –0.11] than the non-pain goals. No significant differences between the pain control goal and the non-pain goals were found for importance, Z = –0.90, *p* = 0.367, control, Z = –0.64, *p* = 0.523, satisfaction with progress, *Z* = –1.84, *p* = 0.07, and value, *Z* = –0.53, *p* = 0.595.

**Table 2:** Descriptive statistics and correlation coefficients between the appraisals of the pain control goal on the one hand, and pain severity, attempts to control pain, meaningfulness of life despite pain, acceptance, and catastrophizing on the other hand.

	*N*	*M* (SD)	Pain Severity (MPI)	Solving pain (PaSol)	Meaning-fulness^b^ (PaSol)	Acceptance (PaSol)	Catastrophizing (PCS)
Importance^b^	73	5.66 (0.67)	0.02	0.26ˆ*	0.12	–0.09	–0.07
Difficulty^a^	73	4.08 (1.57)	0.29ˆ*	0.09	–0.12	–0.20	0.19
Control^b^	73	2.92 (1.67)	–0.08	–0.03	0.20ˆ*	0.24ˆ**	–0.16
Stress^a^	73	3.37 (1.70)	0.07	0.11	–0.18ˆ**	–0.20	0.35ˆ**
Time^b^	73	4.32 (1.51)	0.25ˆ**	0.32ˆ***	0.04	–0.03	0.06
Progress^b^	73	2.74 (1.90)	–0.18ˆ*	–0.17	0.38ˆ***	0.28ˆ**	–0.28ˆ**
Self-Identity^a^	73	3.78 (1.79)	–0.12	0.06	0.17	0.17	–0.20
Value^b^	73	5.51 (0.99)	0.04	0.29ˆ**	0.16	0.10	–0.02

^a^Pearson Correlations; ^b^Kendall’s tau correlations; PaSol, Pain Solutions Questionnaire; PCS, Pain Catastrophizing Scale. ^∗^p < 0.05. **p < 0.01. ***p < 0.001.

**Table 3:** Descriptive statistics and correlation coefficients between appraisals of the non-pain goals (averaged) on the one hand, and pain severity, attempts to solve pain, meaningfulness of life despite pain, acceptance, and catastrophizing on the other hand.

	*N*	*M* (*SD*)	Pain Severity (MPI)	Solving pain (PaSol)	Meaningfulness^b^ (PaSol)	Acceptance (PaSol)	Catastrophizing (PCS)
Importance^b^	73	5.61 (0.47)	0.05	0.08	0.08	–0.01	–0.06
Difficulty^a^	73	3.50 (1.44)	0.17	0.25ˆ*	–0.18ˆ*	–0.30ˆ*	0.37ˆ**
Control^a^	73	3.08 (1.40)	–0.14	–0.21	0.25ˆ**	0.30ˆ*	–0.15
Stress^a^	73	2.92 (1.68)	0.30ˆ**	0.19	–0.32ˆ***	–0.40ˆ***	0.43ˆ***
Time^a^	73	3.95 (1.01)	0.10	0.13	0.16	0.12	0.05
Progress^a^	73	3.21 (1.50)	–0.17	–0.16	0.41ˆ***	0.43ˆ***	–0.39ˆ***
Self-Identity^a^	73	4.37 (1.17)	–0.11	–0.18	0.21ˆ*	0.25ˆ*	–0.21
Value^b^	73	5.62 (0.49)	0.07	0.00	0.12	–0.04	–0.02

*^a^Pearson correlations; ^b^Kendall’s tau correlations; MPI, Multidimensional Pain Inventory; PaSol, Pain Solutions Questionnaire; PCS, Pain Catastrophizing Scale. ^∗^p < 0.05. ^∗∗^p < 0.01. ^∗∗∗^p < 0.001.*

Pearson or Kendall’s tau correlations were calculated to investigate the association between goal appraisals and measures of problem solving, acceptance and catastrophic thinking about pain (see **Tables 2** and **3**). Higher levels of attempts to solve pain (PaSol) were related to rating the pain control goal as more important (*r* = 0.26) and more valuable (*r* = 0.29), and to a higher investment of time in the pain control goal (*r* = 0.32). Acceptance, i.e., acceptance of the insolubility of pain (PaSol) and meaningfulness of life despite pain (PaSol), were found to be positively related to satisfaction with progress in achieving both the pain control goal (*r* = 0.28 and *r* = 0.38, respectively) and non-pain goals (*r* = 0.43 and *r* = 0.41, respectively). Also, acceptance was found to be associated with lower ratings of stress in pursuing non-pain goals (*r* = –0.40). Finally, catastrophizing about pain (PCS) was related to more stress while pursuing the pain control goal (*r* = 0.35) and the non-pain goals (*r* = 0.43), and to lower ratings of satisfaction with progress in achieving both the pain control goal (*r* = –0.28) and the non-pain goals (*r* = –0.39). Catastrophizing was also related to higher ratings of difficulty in achieving non-pain goals (*r* = 0.37).

### Intergoal Relationships

**Table 4** presents the descriptive statistics (mean, *SD*) of the intergoal variables. High levels (response options ≥4) of pain control goal interference were reported by 50% of the participants, 80.15% reported high amounts of pain control goal facilitation, and 59.55% showed high need to control pain before pursuing other goals (necessary condition). Pain goal interference was found to be unrelated to pain goal facilitation (*r* = 0.09). No significant association was found between pain goal facilitation and necessary condition (*r* = 0.08).

**Table 4:** Results of correlational analyses between pain goal interference, facilitation and necessary condition, and solving pain, meaningfulness of life despite pain, acceptance, and catastrophizing.

	*M* (*SD*)	2	3	4	5	6	7	8
1. Pain Goal Interference^a^	3.16 (1.04)	0.08	0.16	0.10	0.23^+^	–0.08	–0.21	0.09
2. Pain Goal Facilitation^b^	4.00 (0.84)		0.08	0.05	0.26^∗∗^	0.04	–0.02	0.05
3. Necessary Condition^a^	3.58 (1.04)			0.16	0.07	–0.20^∗^	–0.31^∗∗^	0.40^∗∗∗^
4. Pain intensity (MPI)	11.78 (2.73)				0.38^∗∗^	–0.32^∗∗^	–0.28^∗^	0.35^∗∗^
5. Solving pain (PaSol)^a^	16.53 (5.62)					–0.08	–0.31	0.34^∗∗^
6. Meaningfulness (PaSol)^b^	20.88 (5.60)						0.55^∗∗^	–0.35^∗∗^
7. Acceptance (PaSol)^a^	8.78 (4.56)							–0.39^∗∗^
8. Catastrophizing (PCS)^a^	23.25 (10.06)							

*^a^Pearson correlations; ^b^Kendall’s tau correlations; PaSol, Pain Solutions Questionnaire; PCS, Pain Catastrophizing Scale. ^+^p = 0.05. ^∗^p < 0.05. ^∗∗^p < 0.01. ^∗∗∗^p < 0.001.*

**Table 4** presents the results of the correlational analyses between intergoal variables on the one hand, and problem solving, acceptance, and catastrophizing. Attempting to solve pain (PaSol) was associated with higher levels of facilitation of pain control goals on non-pain goals (*r* = 0.26). Acceptance of the insolubility of pain (PaSol) and meaningfulness of life despite pain (PaSol), were related to lower levels of necessity of achieving the pain control goal upon pursuing one’s non-pain goals (*r* = –0.31 and *r* = –0.20, respectively). Catastrophizing about pain (PCS) was related to higher levels of necessity of achieving the pain control goal upon pursuing one’s non-pain goals (*r* = 0.40).

## Discussion

This study investigated (1) which goals are spontaneously elicited by ICPs, (2) how ICPs appraise their pain control goals, and whether these differ from non-pain goal appraisals, (3) how the pursuit of pain control goals affects the working on non-pain goals, and (4) whether the goal appraisals and interrelations are related to some key constructs involved in misdirected problem solving, such as attempts to solve pain, acceptance and catastrophic thinking about pain.

Individuals with chronic pains pursued a wide array of personal goals, and although we did not perform an in-depth analysis of their content, they seem to be quite similar to the goals that other individuals report (PPA, Little, 1983). Surprisingly, pain control goals, i.e., goals related to the control and management of pain, were not overly salient in our sample. Only about 40% spontaneously provided a goal related to attempts to control pain. One may have expected a larger percentage. However, several reasons may account for this finding. First, we mainly focused upon the goal to control pain. When categorizing the content of the goals, it became apparent that other pain-related goals were also pursued. The content of about 7% of goals were related to social validation. It seems that next to attempting to try to control pain, being believed by others that the pain is real, is a concern for a subgroup of ICPs (Kool et al., 2013). More research on this largely neglected topic is warranted (Hamilton et al., 2005; De Ruddere and Craig, 2016). Second, the study took place in a research context and not in a clinical setting, in which pain control may be more salient. Relatedly, participants were recruited from a patient association group, and not from a specialist clinic or rehabilitation center. It is likely that not all these individuals show tenacity in trying to solve their pain (Crombez et al., 2008). Third, in the instructions regarding the goal elicitation procedure, ICPs were not prompted with the example of a pain control goal.

Overall, the pattern of results indicates that attempting to control pain is a time-consuming and frustrating enterprise in ICPs (McCracken and Eccleston, 2003, 2005; Eccleston and Crombez, 2007). Participants indicated that their pain control goal was more difficult to achieve, more stressful, and required more of their time than their non-pain goals. They also experienced their pain control goal as less representative for their identity than the non-pain goals. Also, the pattern of correlations is in line with this picture. ICPs who report to be more engaged in solving pain, rated the goal to control pain as more important and valuable, and invested more time in it. Not accepting that pain is insoluble and not believing that life is meaningful despite pain were both associated with being less satisfied with the progress on pain control goals.

A noteworthy finding is that individual differences in attempts to solve pain were not only related to appraisals of the pain control goal, but also to appraisals of the non-pain goals. ICPs who reported to be more engaged in solving pain also reported more difficulties in achieving their non-pain goals. This was also the case for ICPs who do not accept that their pain is insoluble and do not believe that life is meaningful despite pain. The latter two variables were also related to experiencing more stress and less progress during the pursuit of the non-pain goals. There are several avenues to make sense of these data. First, it may be that attempts to solve pain come along with costs in pursuing other goals (Riediger and Freund, 2004). If this is the case one would expect that ICPs also report that working on the pain control goal interferes with the pursuit of non-pain goals. Further data of our study seem to corroborate this interpretation. ICPs who cannot accept pain as insoluble and do not believe that life is meaningful despite pain, report that solving pain is necessary in order to pursue their non-pain goals. Likewise, ICPs who engage more in attempts to solve pain also report more interference of their pain control goal with non-pain goals, although this effect failed to reach statistical significance. Second, it may be that attempts to solve pain are fueled by the interfering effect of pain on goal-related activities. Indeed, in many self-regulation models (Karoly, 1993; Carver and Scheier, 1998; Brandtstädter and Rothermund, 2002) coping and problem solving are triggered by goal interference, an experience that is highly prevalent in ICPs (Karoly and Ruehlman, 2007).

An important objective of this study was to investigate the goal structure or how ICPs experience the relationships between pain and non-pain goals. More specifically, we explored whether working on pain control goals facilitated working on non-pain goals (pain goal facilitation), whether working on pain control goals interfered with working on non-pain goals, and whether achievement of pain control goals is deemed necessary to attain non-pain goals (necessary condition). Despite the extensive piloting with the precise format of the items, some methodological issues remain. First, necessary condition is logically a part of the larger construct of pain goal facilitation. We would then expect a positive association between both, which, however, was not the case. Second, participants reported that the questions on goal interrelations were difficult. It is our impression that overall, the necessity item made more sense than the items on facilitation and interference. Notwithstanding, the pattern of results on the goal structure is intriguing, especially related to the necessity to solve pain in order to pursue non-pain goals. Most notable was our finding that perceiving one’s pain control goal as necessary for achieving other goals, was related to accepting less that pain is insoluble and believing less that life is meaningful despite pain. These findings resonate with the idea of Conditional Goal Setting (CGS; Street, 2002; Street et al., 2007). CGS refers to the mechanism of goal linking, which means that a lower order goal (e.g., to control my pain) is conditionally linked to a higher order value (e.g., be happy). Through goal linking, the lower order goal gains significant importance, and will become difficult to disengage from. As a result, depression and distress may increase (Street, 2002; Street et al., 2007). As such, CGS theory may help explaining tenacious efforts to achieve pain control and resulting distress. Solving pain may become a necessary mean to continue with their life. Individuals may then become stuck in their attempts to solve pain, as an easy solution is not at hand.

Our results challenge some current understandings of catastrophizing about pain, and call for a conceptual broadening. Catastrophizing has been found a robust predictor of pain-related distress and disability in cross-sectional and longitudinal studies (Peters et al., 2007). Traditionally, catastrophizing is defined as an exaggerated negative mental set brought to bear during actual or anticipated pain experience (Sullivan et al., 2001). This is also evidenced in the influential fear-avoidance model. According to this model, those who catastrophize about pain develop erroneous fears about their pain [fear of (re)injury], which lead to avoidance of pain-evoking activities (Vlaeyen and Linton, 2000). Our results indicate that more might be at stake. Individuals who catastrophized about pain reported also elevated levels of stress in pursuing non-pain goals and less satisfaction with progress toward these goals. Those individuals also perceived their pain control goal as a necessary condition for pursuing their non-pain goals. Catastrophizing may thus not be limited to the mere experience of pain, but may extend to the experienced interference of daily goals by pain, possibly resulting in worrying and attempts to problem solving (Eccleston and Crombez, 2007; Crombez et al., 2012).

There are some limitations to this study. First, we adopted a goal and self-regulation perspective (Karoly, 1993; Little, 1998; Crombez et al., 2012) to frame our research and interpret our findings. However, our data are also compatible with other theoretical perspectives, amongst which the psychological flexibility (PF) model (Kashdan and Rottenberg, 2010; McCracken and Morley, 2014). PF could be described as the capacity to be fully in contact with the present moment without needless defense against pain, and to persist or change behavior in function of one’s goals despite pain. For example, our findings on the potential negative effect of seeing pain control as necessary to allow pursuit of other non-pain goals, could be easily translated to PF language. (Hayes et al., 2012; Trompetter et al., 2015; Yu and McCracken, 2016). Second, results of this study are based on cross-sectional data, so we cannot infer causality. The use of moment-to-moment assessment by, for instance, diary approaches and the use of longitudinal designs will be necessary to allow causal inferences. A third limitation relates to sample characteristics. Within this study, we have explored goals in a self-defined chronic pain population, which may not be a representative sample of patients. Fourth, only a limited number of standard goal appraisals were assessed. Other dimensions are possible and may be of further relevance to help understand how patients juggle between the pain and non-pain goals. Fifth, we limited our focus to the goal of controlling pain. However, other pain-related goals, such as being believed by others that the pain is real, may also be at stake and important to assess (Karoly and Jensen, 1987; Hamilton et al., 2005).

## Author Contributions

GC has initiated and developed research ideas and design, and finalized paper. EL was involved in developing research ideas and design, collected and analysed the data, and made a first draft of the paper. LG was involved in developing the research design, and providing feedback on the paper. SD was involved in developing the research design and ideas, and provided feedback on the paper.

## Conflict of Interest Statement

The authors declare that the research was conducted in the absence of any commercial or financial relationships that could be construed as a potential conflict of interest. The reviewer HT and handling Editor declared their shared affiliation, and the handling Editor states that the process nevertheless met the standards of a fair and objective review.
